# Orthodontic care in orthodontic patients during the COVID-2019 pandemic: emergency, emergency response and orthodontic treatment preference

**DOI:** 10.1186/s12903-023-03066-z

**Published:** 2023-06-05

**Authors:** Zeyao Miao, Haijuan Zhang, Yandong Han, Lirong Wang, Shuang Wang

**Affiliations:** 1 Key Laboratory of Shaanxi Province for Craniofacial Precision Medicine Research, College of Stomatology, Xi’an Jiaotong University, Xi’an, Shaanxi China; 2 Department of Orthodontics, College of Stomatology, Xi’an Jiaotong University, Xi’an, Shaanxi China; 3 Clinical Research Center of Shaanxi Province for Dental and Maxillofacial Diseases, College of Stomatology, Xi’an Jiaotong University, Xi’an, Shaanxi China; 4 Department of Epidemiology and Biostatistics, School of Public Health, Xi’an Jiaotong University Health Science Center, Xi’an, Shaanxi China

**Keywords:** COVID-19, Orthodontic emergencies, Orthodontic treatment preference, Pain, Disability

## Abstract

**Background:**

The objective of this study was to investigate the characteristics of emergencies and the requirement for emergency treatment after the suspension of orthodontic appointments. The attitude towards orthodontic treatment preference was evaluated as well, including receiving orthodontic treatment and the preference for orthodontic appliances.

**Subjects and methods:**

An electronic questionnaire was distributed to the patients, including 4 sections: Section 1 – demographic and basic information; Section 2 – the characteristics of emergencies and emergency treatment requirements; Section 3 – the NRS-11 for pain and Manchester Orofacial Pain Disability Scale used to evaluate the intensity of orofacial pain and disability; and Section 4 – attitudes towards receiving orthodontic treatment and appliance preference. Descriptive statistics, Pearson’s chi-square test, Wilcoxon's rank-sum test and stepwise generalized linear model (GLM) were performed with significance set at *P* < 0.05.

**Result:**

Most participants’ (91.61%) follow-up appointments were suspended. The emergency rate and emergency treatment requirements were not different between the fixed appliance (FA) and clear aligner (CA) groups. Patients who reported emergencies (*P* < 0.01) in the FA group (*P* < 0.05) and some emergencies in the FA (*P* < 0.05) suffered worse pain and disability. More FA participants preferred alternative appliances (*P* < 0.05) due to pain and disability (*P* < 0.05).

**Conclusion:**

FA patients’ emergencies caused worse pain and disability when orthodontic appointments were suspended. Pain and disability were not the causes of emergency treatment requirements. The CA group seemed to show a tendency towards orthodontic appliance preference, which was an ideal modality to weather the epidemic, combined with telemedicine.

## Introduction

Coronavirus disease 2019 (COVID-2019) was reported initially in Wuhan, Hubei Province, China, in December 2019 and swept all over the world. The World Health Organization declared COVID-2019 a global pandemic on March 13, 2020, meaning that the disease had worldwide influence by that time. The disease had been reported in more than 210 countries by May 21, 2020. Under the pressure of controlling the outbreak, many countries all over the world announced new policies to prevent it from further spreading [1, 2].

Another local epidemic occurred in Xian, Shan’xi Province, China, on December 9, 2021, which was derived from an imported case and influenced five provinces in China. There were 2050 cases reported in Xian, 2077 cases in Shan’xi and 2119 cases in all five provinces. With a series of anti-epidemic policies announced by the government, the number of cases stopped increasing after 41 days.

During dental treatment, all participants, including patients, dentists and dental assistants, are exposed to aerosols of blood and saliva [3]. Additionally, the routes of epidemic transmission are droplets and close contact transmission [4]. Dental practitioners and patients are facing a higher cross-infection risk [1]. Dental hospitals and clinics in Xian suspended general nonemergency dental treatment and only provided essential emergency dental services for safety. The routine follow-up visits of orthodontics treatment were disturbed, and the duration between appointments was extended [1, 5].

Between orthodontic visits, orthodontic patients might suffer from adverse events, such as pain, discomfort, exposed ends of wires, brackets/bands coming off and loose aligners [6–8]. Unfortunately, as a result of dental hospital and clinic closures, care delivery to active patients was restricted. Finally, the influence of adverse events lasted longer, and patients might suffer from worse pain (which would affect orofacial function, such as eating or speaking). It was reported that there is a strong association between the severity of pain and orofacial function [9].

The main appliances used to treat malocclusion are fixed appliances (FAs) and clear aligners (CAs). The incidence of mucosal injury and orthodontic appliance detachment in FA patients is much higher than that in CA patients. When compared with FA patients, CA patients did not require strict monthly follow-up visits, their chair-time was shorter, and their bonding requirements were lower [10]. At the same time, as CA technology develops, an increasing number of orthodontists treat complex cases, such as extractions, open bite and Class II malocclusion, with it [11]. Comparing CA with other appliances, it seemed that CA together with telemedicine was an ideal modality to weather the epidemic.

Therefore, the objective of this study was to investigate the characteristics of emergencies and the requirements for emergency treatment after the suspension of orthodontic appointments. The attitude towards orthodontic treatment preference was evaluated as well, including receiving orthodontic treatment and orthodontic appliance preference.

## Methods

### Study design and participants

This cross-sectional study was designed to examine orthodontic patients suffering under a 2-month lockdown in Xi’an.

The inclusion criteria of participants were as follows: (1) patients accepting orthodontic treatment in the public hospital; (2) having the ability to understand Mandarin Chinese online; (3) providing informed consent for the use of the questionnaire and the recording of data; (4) finishing all of the items on the questionnaire within the given time; and (5) aged 7 and older. All experimental protocols were established according to the ethical guidelines of the Helsinki Declaration and approved by the ethics committee (Approval no. xjkqll [2022] No. 35). Informed consent was obtained from individuals before they participated in the survey.

All the FA patients were asked to follow up by appointment monthly before the epidemic outbreak. As soon as CA progress began, every CA patient was guided to wear aligners 22 h a day, exchange aligners every 14 days and use aligner chew.

### Questionnaire

We designed an anonymous online questionnaire consisting of 4 sections in Mandarin Chinese via www.wjx.cn with 43 items. In the process of designing it, many professors and scholars majoring in epidemiology and orthodontics gave advice, and many studies were consulted [9, 12–16].

Section 1 gathered demographic and epidemic-related basic information, such as sex, age, education level, hospital location, orthodontic application, time since the last appointment, reasons for postponing, and contact between dentists and patients, with 14 items.

Section 2 had 20 items that investigated the orthodontic problems and emergencies reported by patients during the lockdown, such as “debonded bracket” and “attachment drop”. The methods that they undertook to resolve emergencies and the intention to receive emergency treatment in public hospitals were also assessed.

Two mature scales were used to investigate orofacial pain and disability in Section 3: (1) the Numerical Rating Scale (NRS-11) of pain [17] is a segmented numerical scale on a horizontal line with 11 numbers from 0 to 10 – 0 = no pain to 10 = the worst pain the patients can imagine; the scale was used to measure the average pain of patients’ orofacial region; and (2) the Manchester orofacial pain disability scale (MOPDS) [9, 18] can evaluate the disability related to the intensity of the pain from the orofacial region in the past month. The top part of this questionnaire consists of two questions about orofacial pain for more than 24 h in the past month and whether the patient sought professional advice. The other part consisted of 26 questions and was divided into two components: 7 questions for physical disability and 19 questions for psychological disability. Every question used a 3-item Likert scale: 0 = none of the time, 1 = on some days, and 2 = on most/every day; and the final score ranged from 0 to 52.

Section 4 focused on the participants’ attitudes about receiving orthodontic treatment and their orthodontic appliance preferences after the two-month lockdown experience.

The Cronbach’s alpha of this questionnaire was 0.937, which showed high reliability. KMO and Bartlett’s test were used to test the validity, and the KMO value was 0.959, which showed high validity of the study data.

### Data collection

Twelve orthodontists from the orthodontic department were invited to participate in the project. The distribution and collection of the questionnaire were available online from January 24 to January 28. To reduce recall bias and increase authenticity, we distributed the questionnaires immediately after lifting the lockdown and collected them in four days.

### Statistical analysis

We used the mean, median, range and interquartile range in descriptive statistics. NRS-11 and MOPDS scores were recorded to evaluate pain and disability. Pearson’s chi-square test was used to compare the differences in emergencies and the preferences for orthodontic treatment in the two groups (FA and CA). Wilcoxon’s rank-sum test was used to compare the differences in NRS-11 and MOPDS scores between different groups for skewed distribution data. Then, stepwise regression was used in the generalized linear model (GLM) to investigate the relationship between discomfort (pain and disability) and various factors when controlling for potential confounders. The level of statistical significance was set at *P* < 0.05. All analyses were performed using IBM SPSS Statistics software (version 18; IBM Corp, Armonk, NY, USA).

## Results

### Participant characteristics

A total of 200 online questionnaires were distributed to the patients accepting orthodontic treatment, and 154 valid questionnaires were collected. The mean age was 21.54 years old (SD 7.07). Eighty-one (52.6%) participants were younger than 23. A total of 110 (71.43%) were female, and 144 (93.5%) accepted treatment in Xian. Only 12 (8.39%) accepted orthodontic treatment within 1 month, and 52 (36.36%) had not visited for more than 2 months. A total of 104 (79.39%) stopped treatment for fear of the spread of COVID-2019. A total of 83 (58.04%) communicated with their dentists, the majority of participants (92.77%) used mobile phone apps (WeChat), and none of them used telemedicine (Table 1).

**Table 1:** Basic information of participants

		n	%
Age	≤ 23	81	52.6
	> 23	73	47.4
Sex	Male	44	28.6
	Female	110	71.4
Educational level	Primary school and lower	13	8.4
	Junior high school	19	12.3
	Senior high school	17	11.1
	Junior college	11	7.1
	Undergraduate	70	45.5
	Graduate or Higher	24	15.6
Hospital	In Xi’an	144	93.5
	Outside of Xi’an	10	6.5
Type of appliances	Fixed appliances	89	57.8
	Clear aligners	54	35.1
	Removable appliances	11	7.1
Duration from the last appointment	Within 1 mo	12	8.4
	1–1.5 mo	35	24.5
	1.5–2 mo	44	30.8
	More than 2 mo	52	36.3
Reason for postponing	Clinic was closed	71	54.2
	I was afraid of the spread of COVID-2019	104	79.4
	I was out of the city	47	35.9
	Others	40	30.5
Communicated	Yes	83	58.1
	No	60	41.9
Communication channel	Call	13	15.7
	Mobile phone application (Wechat)	77	92.8
	Telemedicine (Smart Good Hospital APP)	0	0.0
	Sent E-mail	0	0.0
	Others	2	2.5

### Emergency and emergency treatment requirement

**Fig. 1:**
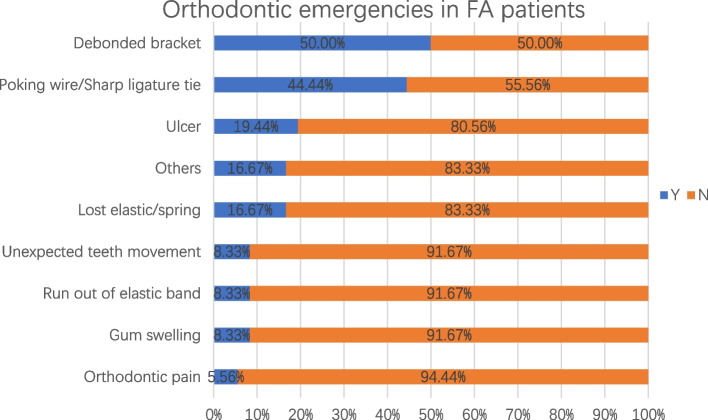
Orthodontic emergencies in FA patients

In the FA group, 36 (40.4%) patients reported emergency treatment, and 12 (33.3%) of them wanted emergency treatment. The most common trouble reported was “Debonded brackets” (50%), and the others are presented in Fig. 1.

**Fig. 2:**
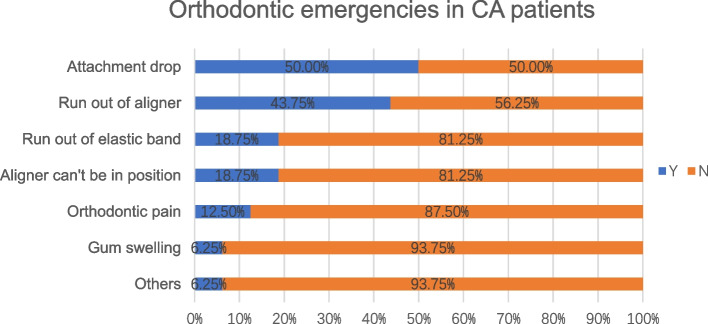
Orthodontic emergencies in clear aligner patients

For the CA patients, 16 (29.63%) reported emergency treatment, and 7 (43.75%) of them required emergency treatment. The most common emergency was “attachment drop” (50%), and the others are shown in sequence in Fig. 2.

**Fig. 3:**
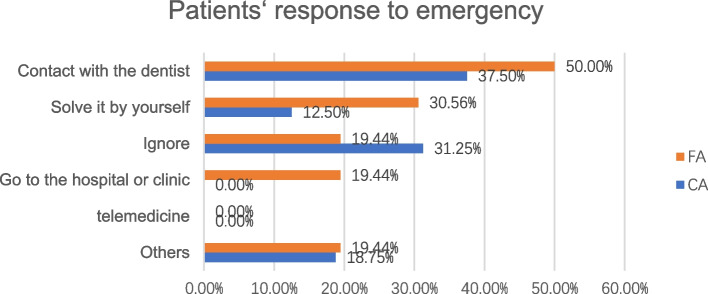
Patients’ responses to emergencies

In response to emergencies, most patients contacted their dentists during the lockdown, and none of them used telemedicine. The response to emergencies between the two appliances was not significantly different and the details of response in the two groups were showed in Fig. 3. During the lockdown, 64 (44.76%) worried about the effect of treatment, and 61 (42.66%) were anxious about their duration of treatment.

The hierarchical Comparison of the emergencies in the two orthodontic types was showed in Table 2. There were no significant differences in the incidence or proportions of emergencies and emergency treatment requirements between the FA and CA groups (Table 3).

**Table 2:** Hierarchical Comparison of the emergencies in the two orthodontic types, (%)

	FA	CA
Debonded brackets	50%	-
Attachment drop	-	50%
Poking wires/Sharp ligature ties	44.44%	-
Ran out of aligners	-	43.75%
Ulcers	19.44%	-
Ran out of elastic bands	8.33%	18.75%
Aligner cannot be in position	-	18.75%
Lost elastics/springs	16.67%	-
Orthodontic pain	5.56%	12.50%
Unexpected tooth movement	8.33%	-
Gum swelling	8.33%	6.25%

**Table 3:** Differences between orthodontic types in emergency conditions, n (%)

		FA	CA	χ^2^	P
Emergency	Y	36(40.4)	16(29.6)	1.7	0.192
	N	53(59.6)	38(70.4)		
Emergency treatment requirement	Y	12(33.3)	7(43.8)	0.518	0.472
	N	24(66.7)	9(56.2)		

### Treatment preference

Nineteen (13.29%) expressed a negative attitude when asked whether they would accept orthodontic treatment assuming that treatment had not yet started. The willingness to accept orthodontic treatment between the two groups was not significantly different (Table 4). Thirteen (16.7%) FA patients and 2 (4.3%) CA patients preferred the alternative appliance, the most common reason for choosing FA was therapeutic effect (53.5%), and that for choosing CA (37.1%) was aesthetics. The proportion of participants who preferred the alternative appliance in FA was higher than that in CA (χ^2^ = 4.129, *P* < 0.05) (Table 4).

**Table 4:** Differences between orthodontic types in orthodontic treatment preference, n (%)

		FA	CA	χ^2^	P
Receive orthodontic	Y	78(87.6)	46(85.2)	0.176	0.625
	N	11(12.4)	8(14.8)		
Appliances preference	Original appliance	65(83.3)	44(95.7)	4.129	0.042
	Alternative appliance	13(16.7)	2(4.3)		

### Pain and disability

The median pain intensity on the NRS-1 1 was 2 (range: 1–9). No significant differences were found between the different sexes in the intensity of pain or different lengths of postponements. Participants who reported emergencies (M = 3, P25, P75 = 1.25,5 vs. M = 2, P25, P75 = 1,4. Ζ = -2.892, *P* < 0.01) and the FA patients (M = 2, P25, P75 = 1,4 vs. M = 1, P25, P75 = 1,4.Ζ = -1.989, *P* < 0.05) had worse pain (Table 5). After adjusting the GLM stepwise, only patients with emergencies felt worse pain (*P* < 0.01) in multivariable analysis (Table 6).

**Table 5:** Difference in pain/disability in emergency conditions

		NRS-11			MOPDS		
		M (P25, P75)	Z	P	M (P25, P75)	Z	P
Sex	Male	2(1,3)	-0.97	0.332	2(0,9.5)	-0.196	0.844
	Female	2(1,4)			3(0,7.5)		
Type	FA	2(1,4)	-1.989	0.047	3(0,10)	-2.41	0.016
	CA	1(1,4)			1(0,4.5)		
Emergency	Y	3(1.25,5)	-2.892	0.004	5(0,11)	-2.871	0.004
	N	2(1,4)			2(0,4)		
Delay	< 1mo	2(1,4)	1.370	0.713	3(1,8.75)	1.827	0.609
	1 mo-1.5 mo	2(1,5)			3(0,10)		
	1.5 mo-2 mo	2(1,4)			2(0,6.25)		
	> 2 mo	2.5(1,4)			3(0,7.75)		
Emergency requirement	Y	3(2,5)	-0.444	0.657	7(0.5,12)	-0.98	0.327
	N	3(1,6)			3(0,10)

**Table 6:** Difference in pain/disability in emergency-associated factors with GLM

	pain				disability			
	B(SE)	Wald CI	Wald χ²	P	B(SE)	Wald CI	Wald χ²	P
Sex								
Female	0.458(0.3378)	-0.204,1.120	1.841	0.175	0.6261(0.1028)	-1.536,2.787	0.322	0.57
Male	Reference				Reference			
Type								
CA	-0.326(0.3139)	-0.941,0.290	1.076	0.3	-1.456(1.0248)	-3.465,0.552	2.019	0.155
FA	Reference				Reference			
Emergency								
N	-1.026(0.3208)	-1.654, -0.397	10.22	0.001	-3.466(1.0474)	-5.518, -1.413	10.95	0.001
Y	Reference				Reference			
Delay								
>2 mo	0.039(0.5832)	-1.104,1.182	0.004	0.947	-1.723(1.9039)	-5.455,2.008	0.819	0.365
1.5 mo-2 mo	-0.435(0.5945)	-1.600,0.731	0.535	0.465	-2.168(1.9408)	-5.972,1.636	1.248	0.264
1 mo-1.5 mo	-0.174(0.6076)	-1.365,1.017	0.082	0.774	-0.597(1.9835)	-4.485,3.290	0.091	0.763
<1 mo	Reference				Reference			

The “poking wires/sharp ligature ties” (M = 4.5, P25, P75 = 2.25,6 vs. M = 2, P25, P75 = 1,4) and “ulcer” (M = 5, P25, P75 = 5,6 vs. M = 3, P25, P75 = 1.5,4) groups had higher pain scores in the rank-sum test (Ζ = -2.532, *P* < 0.05. Ζ = -2.834, *P* < 0.05) in Table 7. After GLM analysis, only “ulcer” had a higher pain score (*P* < 0.05) when controlling for other factors, such as “orthodontic pain” and “poking wires” (Table 8). Patients reporting “aligner cannot be in position” (M = 1 vs. M = 3, Ζ = -2.08, *P* < 0.05) had lower pain in Table 7. The GLM established with various CA emergencies showed that patients reporting “aligner cannot be in position” (*P* < 0.001), “attachment drop” (*P* < 0.01), and “ran out of aligners” (*P* < 0.01) had lower pain scores, while those reporting “orthodontic pain” (*P* < 0.001) had higher scores (Table 9).

**Table 7:** Differences in pain/disability in orthodontic treatment preference

	preference	NRS-11			MOPDS		
		M (P25, P75)	Z	P	M (P25, P75)	Z	P
Receive orthodontic treatment	Y	2(1,4)	-2.385	0.017	2(0,6)	-2.751	0.006
	N	3(2,6)			9(0,20)		
FA	FA	2(1,4)	-2.538	0.011	2(0,5.25)	-2.651	0.008
	CA	4(2.5,6)			8(3,14)		
CA	FA	1(\)	-0.388	0.698	6(\)	-1.986	0.047
	CA	1(1,3)			1(0,3)

**Table 8:** Association between pain/disability and reported emergencies

		NRS-11			MOPDS		
		M (P25, P75)	Z	P	M(P25,P75)	Z	P
Poking wires/Sharp ligature ties	Y	4.5(2.25,6)	-2.532	0.011	11.5(5,13.75)	-2.826	0.005
	N	2.5(1,4)			2.5(0,9.75)		
Debonded brackets	Y	2.5(1,4.25)	-1.651	0.104	8.5(0.75,11.25)	-0.064	0.963
	N	4(2,6)			6.5(0,13.25)		
Lost elastics/springs	Y	3.5(2,4.5)	-0.279	0.780	5(0,11.5)	-0.578	0.563
	N	3(1.75,5)			8(0.75,12)		
Ran out of elastic bands (FA)	Y	4(\)	-1.305	0.192	2(\)	-0.866	0.386
	N	3(2,5)			8(0.5,12)		
Gum swelling	Y	3(\)	-0.493	0.622	5(\)	-0.549	0.583
	N	3(2,5)			8(0,11.5)		
Orthodontic pain (FA)	Y	3.5(\)	-0.245	0.807	7(\)	-0.035	0.972
	N	3(2,5)			8(0.75,11.25)		
Unexpected tooth movement	Y	4(\)	-1.305	0.192	17(\)	-1.068	0.285
	N	3(2,5)			8(0.5,11)		
Ulcers	Y	5(5,6)	-2.834	0.005	12(4,13)	-1.633	0.102
	N	3(1.5,4)			5(0,11)		
Aligner cannot be in position	Y	1(\)	-2.08	0.038	0(\)	-1.713	0.087
	N	3(1.5,5.5)			5(0.5,17)		
Attachment drop	Y	2(1,5.25)	-0.271	0.798	4(0.25,10)	-0.268	0.798
	N	3(1,4.75)			2(0,18.75)		
Ran out of aligners	Y	2(1,3)	-1.037	0.3	3(1,6)	-0.054	1
	N	3(1,7)			2(0,23)		
Orthodontic pain (CA)	Y	7.5(\)	-2.128	0.033	23(/)	-2.103	0.035
	N	2(1,3.25)			2.5(0,6.25)		
Ran out of elastic bands (CA)	Y	3(\)	-0.416	0.677	7(\)	-0.754	0.451
	N	2(1,4.5)			3(0,14.5)

**Table 9:** Difference in pain/disability in FA emergencies after stepwise GLM

	Pain				Disability			
	B(SE)	Wald CI	Wald χ²	P	B(SE)	Wald CI	Wald χ²	P
Poking wires								
Y	0.828(.6420)	-0.430，2.087	1.665	0.197	6.447(2.2750)	1.988,10.906	8.03	0.005
N	Reference				Reference			
Orthodontic pain								
Y	-0.545(1.1847)	-2.867，1.777	0.212	0.645	-0.067(4.1983)	-8.296,8.161	0	0.987
N	Reference				Reference			
Ulcers								
Y	1.808(0.8203)	0.200，3.416	4.859	0.028	-1.419(2.9067)	-7.116,4.278	0.238	0.625
N	Reference				Reference			

Additionally, the participants who maintained negative attitudes towards accepting orthodontic treatment (M = 2, P25, P75 = 1,4 vs. M = 3, P25, P75 = 2,6. Ζ = -2.385, *P* < 0.05) had higher pain scores, and the participants preferred CA over FA (M = 2, P25, P75 = 1,4 vs. M = 4, P25, P75 = 2.5,6. Ζ = -2.538, *P* < 0.05) (Table 10).

**Table 10:** Difference in pain/disability in CA emergencies after stepwise GLM

	Pain				Disability			
	B(SE)	Wald CI	Wald χ²	P	B(SE)	Wald CI	Wald χ²	P
Aligner cannot be in position								
Y	-3.817(0.9046)	-5.590, -2.044	17.803	0	-8.690(3.4074)	-15.368, -2.012	6.505	0.011
N	Reference				Reference			
Attachment drop								
Y	-1.901(0.6397)	-3.155, -0.648	8.836	0.003	-5.141(2.4094)	-9.863, -0.419	4.553	0.033
N	Reference				Reference			
Ran out of aligners								
Y	-2.493(0.7386)	-3.941, -1.045	11.392	0.001	-5.296(2.7821)	-10.749,0.157	3.623	0.057
N	Reference				Reference			
Orthodontic pain								
Y	3.951(1.0676)	1.858,6.043	13.694	0	17.070(4.0214)	9.189,24.952	18.019	0
N	Reference				Reference			

The median disability score was 2 (range 0–25). The most common physical disabilities were “I cannot eat hard foods” (53.14%) and “I take longer to finish my meals” (44.06%), and the highest scores in psychosocial disabilities were for “I have had to take time off work” (23.08%) and “I find it difficult to talk for long periods of time” (21.75%), followed by “I am irritable, angry, and easily frustrated” (21.68%). Similar characteristics were found in most results on the MOPDS and NRS-11 for pain. However, in the GLM, patients reporting “poking wires” reported worse disability (*P* < 0.01) in the FA group; those reporting “aligner cannot be in position” (*P* < 0.05) and “attachment drop” (*P* < 0.05) suffered less disability, and those reporting “orthodontic pain” (*P* < 0.001) reported higher disability scores in the clear aligners group (Tables 8 and 9).

There was a significant association in the linear regression model between the intensity of pain and disability score (τ = 7.525, *P* < 0.001) with the Equation y = 1.753 (95% confidence interval 1.293–2.214) x + 0.084.

## Discussion

Coronavirus disease 2019 (COVID-2019) has been rampant all over the world since 2019. Therefore, many countries have been influenced by the virus, and the pandemic led to a huge burden on medical resources. During dental procedures, respiratory droplets and close contact are regarded as the main transmission routes for the virus [3, 4]. In many countries, such as Italy and China, governments declared highest-level health emergencies and published many policies, such as asking residents to stay at home and suspending normal medical practice. Anti-epidemic measures and the fear of the virus prolonged the interval of orthodontic follow-up appointments, which might have increased the risk of orthodontic emergencies and affected patients’ attitudes about receiving orthodontic treatment, as well as orthodontic appliance preferences [6–8].

In our study, female patients accounted for 71.43% of all participants because they tended to accept orthodontics treatment [19, 20]. Similar to the studies by Zheng and Turkistani KA [9, 21], the pain intensity between male and female patients was not different (*P* > 0.05). Most participants’ (91.41%) normal follow-up appointments were suspended for the lockdown, and some of them (36.3%) could not visit for more than two months. Although the interval was prolonged, the negative effects on pain and disability did not appear for that the suspension of appointments was short.

FA patients had equal opportunity for suffering emergency compared with CA patients (*P* > 0.05). Worse pain was caused by “ulcers” (19.44%) and “poking wires/sharp ligature ties” (44.44%) in the FA group. When controlling for other confounding factors, ulcers might be the main cause of pain. In contrast, “poking wires/sharp ligature ties” were the main causes of orofacial disability, and ulcers did not affect oral activities, such as eating and speaking [22]. Participants reporting “attachment drop” (50%), “ran out of aligners” (43.75%) and “aligner cannot be in position” (18.75%) had relatively mild discomfort. The reason for this phenomenon was decreased controlling force inflicted on the teeth, which might make aligners less effective and prolong the course of treatment. Comparing with CA emergencies, the FA emergencies tended to cause greater discomfort, and FA patients reported higher pain intensity (*P* < 0.05) and MOPDS (*P* < 0.05) scores. More comfort should be given to FA patients when the appointments were suspended for long time, and “ulcers” and “poking wires/sharp ligature ties” would be the major concern.

The requirement for emergency treatment between the two appliances showed no differences. The pain (*P* < 0.005) and disability (*P* < 0.005) scores in patients who reported emergency were significantly higher, but the patients who required emergency treatment did not suffer worse pain and disability. Eight-one FA patients’ (91.01%) routine appointments were suspended and their orthodontic progresses were interrupted. Sixteen CA patients (29.10%) reported emergency like “attachment drop”, “ran out of aligners” and “aligner cannot be in position”, which might reduce the efficiency of aligners and prolong the treatment course [23]. Thus, the factor that actually led to orofacial pain and disability was emergencies. Anxiety about the treatment effect (68.53%) and course of treatment (85.31%), rather than the pain, disability and appliance types, might have led to emergency treatment requirements. A large number of patients (79.39%) stopped visiting their dentists for fear of COVID-19, which might be another contributing factor to emergency requirements. We discovered that each missed appointment added 1.09 months to the treatment time from Beckwith et al. [24], which had less influence on the whole course of treatment. Therefore, orthodontics emergency treatment might not be necessary in the context of the pandemic. FA patients could solve these emergencies with orthodontic wax and ulcer patches during lockdown. Additionally, patients with clear aligners could wear old aligners and receive new aligners by delivery, which might be a solution of keeping orthodontic progress when visits are suspended. Also, Xiong X reported that patients suffering from epidemics had higher odds of mental distress with longer isolation from society. The delayed time interval from the last dental visit was a factor associated with mental problems [16]. Dentists should pay more attention to their patients’ mental health and find an effective method to communicate and relieve patients’ anxiety.

With the experience of suspending orthodontic appointments during the pandemic, the willingness to accept orthodontic treatment (86.71%) was reduced for pain and disability (*P* < 0.05), assuming that the participants had not yet started orthodontic procedures. Although orofacial pain and disability influenced the attitude of accepting orthodontic treatment, the difference in participants’ discomfort between the FA and CA groups was so fine that the willingness to accept orthodontic treatment was similar between the two groups.

The preference for appliances in the FA and CA participants was different. A significant change was observed in the FA participants’ appliance preferences compared with the CA group (*P* < 0.05), and participants in the FA group who preferred CA suffered more pain (*P* < 0.05) and disability (*P* < 0.01). Therefore, it would seem that the FA group tended to select alternative orthodontic appliances (CA). The worse pain and disability caused the change in FA participants’ appliance preferences. Additionally, Xiong X [16] reported that patients who used lingual appliances and clear aligners were less anxious about the course of treatment for invisible characteristics, which might be another reason for the preference for CA. Reserving sufficient aligners would reduce the harmful effects of emergencies and the suspension of appointments. Telemedicine is an ideal contact method between dental patients and dentists for maintaining better orthodontic conditions and avoiding unnecessary close contact [25–29]. Telemedicine and CA combined reduced chair time, bonding requirements and risks of cross-infection, so it was an ideal form of orthodontic therapy during the spread of the pandemic [5, 25].

This study had some limitations. The sample size was determined by the small range of the COVID-19 epidemic in a city. The lockdown was soon lifted so that the suspension of appointments was for only 2 months. The questionnaire survey that we designed might inevitably have incurred recall bias.

## Conclusions

1. When suspending orthodontic appointments, emergencies in FA patients caused worse pain and disability. More comfort should be given to FA patients when the appointments were suspended for long time.
2. Pain and disability were not the causes of emergency treatment requirements. Guiding patients in resolving emergencies by themselves and caring about the mental issues that arise due to suspending orthodontic treatment might be beneficial, as well as avoiding unnecessary orthodontic emergency treatment. Also, CA combined with delivery seems to be a solution of keeping orthodontic progress when visits are suspended.
3. The CA seemed to show a tendency towards being orthodontic appliance preference after the experience of suspending orthodontic appointments during the pandemic, and it was an ideal modality to weather the epidemic and reduce the spread of virus, combined with telemedicine.

## Data Availability

The datasets used and/or analysed during the current study are available from the corresponding author on reasonable request.
